# CHEMICAL EXPOSURES: More Iodine or Less Perchlorate?

**Published:** 2010-07

**Authors:** Rebecca Renner

**Affiliations:** **Rebecca Renner**, PhD, of Williamsport, PA, is a long-time contributor to *EHP* and *Environmental Science & Technology*. Her work has also appeared in *Scientific American*, *Science*, and Salon.com

Perchlorate is believed to block uptake of iodine into the thyroid, eventually resulting in the decreased production of the thyroid hormones thyroxine and triiodothyronine. But a science review of perchlorate concludes that reducing the risk of mental deficits in children whose mothers are exposed to the chemical may be achieved most efficiently by correcting the iodine deficiency that occurs in roughly a third of U.S. women of child-bearing age—not by reducing perchlorate intake.^1^

The review is a first for the U.S. Environmental Protection Agency (EPA) Office of Inspector General (OIG), which primarily conducts audits, evaluations, and investigations of the EPA and its contractors to promote economy and efficiency, and to prevent and detect fraud, waste, and abuse. But rather than resolving controversy over the risk characterization of perchlorate, the review appears instead to be further fueling it.^2^

In comments offered in response to the review, the Environmental Working Group wrote that the OIG had used the review to justify their endorsement of the Bush Administration’s failure to set a drinking water standard perchlorate, which pollutes the drinking water of an estimated 20–40 million people nationwide.^3^ But Purnendu Dasgupta, an analytical chemist at the University of Texas, Arlington, applauds the OIG for stepping in to address a major public health gap. “The continued brouhaha about perchlorate alone, whether by activists or protectionists, merely acts as a smokescreen,” he says. “We have urgent problems about iodine nutrition; the preoccupation with perchlorate alone is obscuring the fact that we are gambling with the intellectual future of the next generation at our peril.”

Perchlorate is thought to affect thyroid function by blocking uptake of iodine, an essential component of thyroid hormones, which orchestrate brain development. Other chemicals—in particular, thiocyanate (found in tobacco smoke and cruciferous vegetables) and nitrate (found in leafy vegetables, processed meats, and some contaminated water supplies)—act in a similar way. Too little iodide also has the same effect. The OIG considered all four of these factors in its cumulative risk assessment, a type of assessment that looks at the public health risk arising from multiple, combined stressors.

By attempting a more holistic cumulative assessment, OIG says it is at the vanguard of governmental agencies in following recommendations from several recent governmental advisory committees.^2^ House and Senate draft versions of chemical regulation reform bills also call for cumulative risk assessments. Risk assessment specialists generally applaud this innovative aspect of the OIG effort. But many comments on the report referred to the lack of peer review, failure to consider major studies, failure to specifically consider the risk of perchlorate exposure to infants, and an excessive reliance on one *in vitro* study^4^ that estimated the relative potencies of the different thyroid stressors in terms of their ability to block iodine uptake.

The OIG hired consultancy ICF International to conduct a technical review of the assessment. ICF International broadly endorsed the OIG’s cumulative risk assessment approach, but recommended the use of more recent peer-reviewed human studies, in particular a 2006 study^5^ that found a statistically significant association between changes in thyroid function to levels of perchlorate exposure roughly an order of magnitude lower than those in previous studies of people exposed to perchlorate.

The Environmental Working Group contends ICF International had a potential conflict of interest because the firm has consulted for federal agencies, military contractors, and other entities responsible for perchlorate pollution in drinking water supplies, all of whom “have vigorously opposed strong public health standards for perchlorate.”^3^ The Massachusetts Department of Environmental Protection raised similar concerns. But the OIG contends ICF International was selected as the best qualified bidder under federal guidelines.^2^

Other questions revolve around data suggesting perchlorate may have additional mechanisms of action beyond its ability to inhibit iodine uptake.^6^ “Although the OIG study is informative with respect to cumulative impacts at the level of thyroidal iodine uptake, the potential existence of additional mechanisms of action should temper conclusions regarding appropriate perchlorate exposure limits, especially where the iodine uptake inhibition estimates are derived from an *in vitro* model that does not reflect the complexity of *in vivo* thyroid function, effects, and responses,” says toxicologist C. Mark Smith of the Massachusetts Department of Environmental Protection.

Adam Finkel, a member of the National Research Council committee that evaluated EPA risk assessment protocols,^7^ notes moreover that cumulative risk assessments such as this could end up yielding questionable policy. “Advocates for holistic risk assessments assumed the point to be that you can make a stronger case for reducing pollutant X if you see it in context of all the other things also adding to the burden of disease Y—but this report turns that logic on its head and says essentially that when you see the whole picture, you see a reason to ignore the pollutant and work on the other things,” he explains.

The conclusions of the review conflict with risk assessments conducted by states such as California and Massachusetts, which have adopted health recommendations more stringent than the current EPA reference dose for perchlorate of 0.0007 mg/kg/day (total intake from both water and food). “Although improving iodine nutrition is an important public health issue itself, it is an incomplete response to perchlorate drinking water contamination,” Smith says. “Infants are the population of greatest concern identified in the Massachusetts risk assessment, but the OIG assessment doesn’t adequately address their demonstrated potential for significant perchlorate exposure and risk.”

“It’s great that this cumulative assessment looks more broadly and seeks to consider possible risk management solutions early in the assessment process,” says Finkel. “But while adding iodide may be the most efficient solution, that is not for the risk assessor to prejudge—we need a document that lays out the costs and benefits of alternative approaches, not one that trivializes the environmental risk because there may be a ‘supply side’ way of sidestepping it.”

Jonathan Levy, who also was a member of the panel that evaluated EPA risk assessment protocols,^7^ agrees. “Our NAS committee recommendations would argue that the presence of multiple stressors would imply that health effects would be anticipated at low dose of perchlorate,” he says. “The fact that other stressors have greater effects is an interesting observation, but we explicitly stated that this should not be the primary output of cumulative risk assessment.”
